# Diffuse bone marrow metastasis of cancer cells mimicking hematologic malignancy in a case of rhabdomyosarcoma

**DOI:** 10.1002/jha2.137

**Published:** 2020-11-29

**Authors:** Chao‐Hung Wei, Chien‐Chin Lin, Jen‐Chieh Lee, Hwei‐Fang Tien

**Affiliations:** ^1^ Division of Hematology, Department of Internal Medicine National Taiwan University Hospital Taipei Taiwan; ^2^ Department of Laboratory Medicine National Taiwan University Hospital Taipei Taiwan; ^3^ Department of Pathology National Taiwan University Hospital Taipei Taiwan

A 29‐year‐old female with clinical manifestation of multiple ecchymosis, menorrhagia, bicytopenia, and leukoerythroblastosis was referred to the Hematology Division of this Medical Center from a local hospital. Her hemoglobin level on arrival was 6.0 g/dL, platelet count, 18 × 10^9^/L, and white blood cell count, 6.09 × 10^9^/L with 6% of blasts, 10% of promyelocytes, 10% of myelocytes, 21% of neutrophils, and presence of normoblasts. Due to high suspicion of hematologic disease, a bone marrow study was performed. The marrow film showed diffuse infiltration of large and bizarre immature cells with basophilic cytoplasm and cytoplasmic vacuoles (top left), mimicking high‐grade hematologic malignancy. Cytochemical stainings including myeloperoxidase, chloroacetate esterase, and nonspecific esterase were all negative. Flow cytometry showed bright CD56 expression, but negative for CD45 and all other hematopoietic stem/progenitor, myeloid, and lymphoid cell markers. Bone marrow cytogenetics analysis revealed t(2;13)(q35;q14). The initial histopathologic exam of the bone marrow biopsy specimen revealed malignant round cells infiltration, which were focally positive for desmin, while negative for several other lineage specific markers. No definite diagnosis could be made. Whole body positron emission tomography showed diffused high‐intensity signals over axial and appendicular skeletons and bilateral adnexae (bottom left). Laparoscopic right salpingo‐oophorectomy was carried out for more accurate diagnosis (bottom right), and the histopathological examination confirmed the diagnosis of rhabdomyosarcoma, alveolar type. Fluorescence in situ hybridization study was positive for *FOXO1* gene break‐apart, indicating the presence of a translocation (top right), which was specific for rhabdomyosarcoma, alveolar type, in tumor cells. She was transferred to the Oncologic Department for further chemotherapy.[Fig jha2137-fig-0001]

**FIGURE 1 jha2137-fig-0001:**
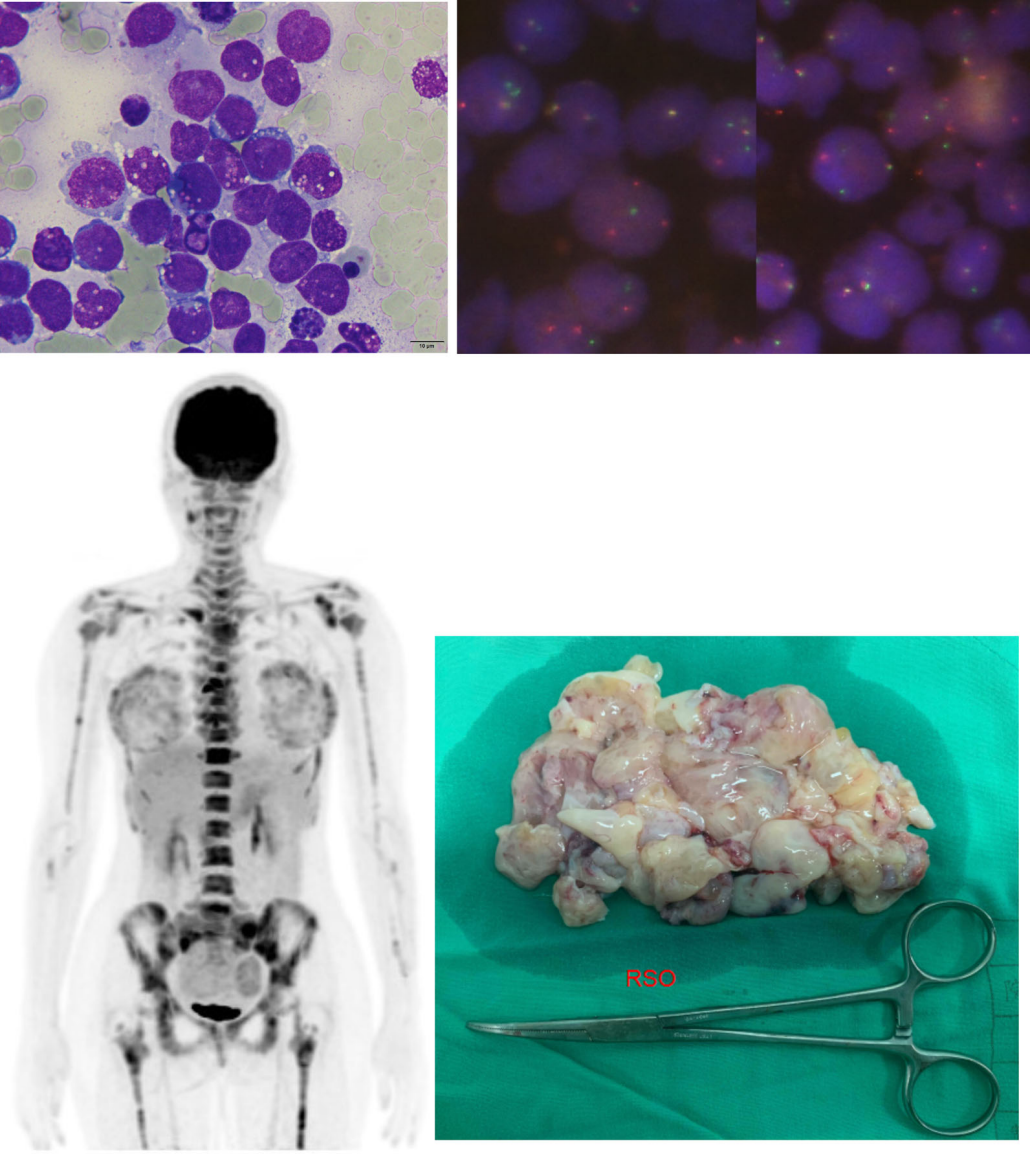
**Top left**. The marrow film showed diffuse infiltration of large and bizarre immature cells with basophilic cytoplasm and cytoplasmic vacuoles. **Bottom left**. Whole body positron emission tomography showed diffused high‐intensity signals over axial and appendicular skeletons and bilateral adnexae. **Bottom right** Laparoscopic right salpingo‐oophorectomy was carried out for more accurate diagnosis. There was a papillary ovarian tumor without intact capsule. **Top right**. Fluorescence in situ hybridization study was positive for FOXO1 gene break‐apart, indicating the presence of a translocation.

